# NMDA Receptor Function During Senescence: Implication on Cognitive Performance

**DOI:** 10.3389/fnins.2015.00473

**Published:** 2015-12-16

**Authors:** Ashok Kumar

**Affiliations:** Department of Neuroscience, Evelyn F. and William L. McKnight Brain Institute, University of FloridaGainesville, FL, USA

**Keywords:** aging, hippocampus, oxidative stress, NMDA receptor, GluN2A, GluN2B, learning, spatial memory

## Abstract

N-methyl-D-aspartate (NMDA) receptors, a family of L-glutamate receptors, play an important role in learning and memory, and are critical for spatial memory. These receptors are tetrameric ion channels composed of a family of related subunits. One of the hallmarks of the aging human population is a decline in cognitive function; studies in the past couple of years have demonstrated deterioration in NMDA receptor subunit expression and function with advancing age. However, a direct relationship between impaired memory function and a decline in NMDA receptors is still ambiguous. Recent studies indicate a link between an age-associated NMDA receptor hypofunction and memory impairment and provide evidence that age-associated enhanced oxidative stress might be contributing to the alterations associated with senescence. However, clear evidence is still deficient in demonstrating the underlying mechanisms and a relationship between age-associated impaired cognitive faculties and NMDA receptor hypofunction. The current review intends to present an overview of the research findings regarding changes in expression of various NMDA receptor subunits and deficits in NMDA receptor function during senescence and its implication in age-associated impaired hippocampal-dependent memory function.

## Introduction

**Figure 1:**
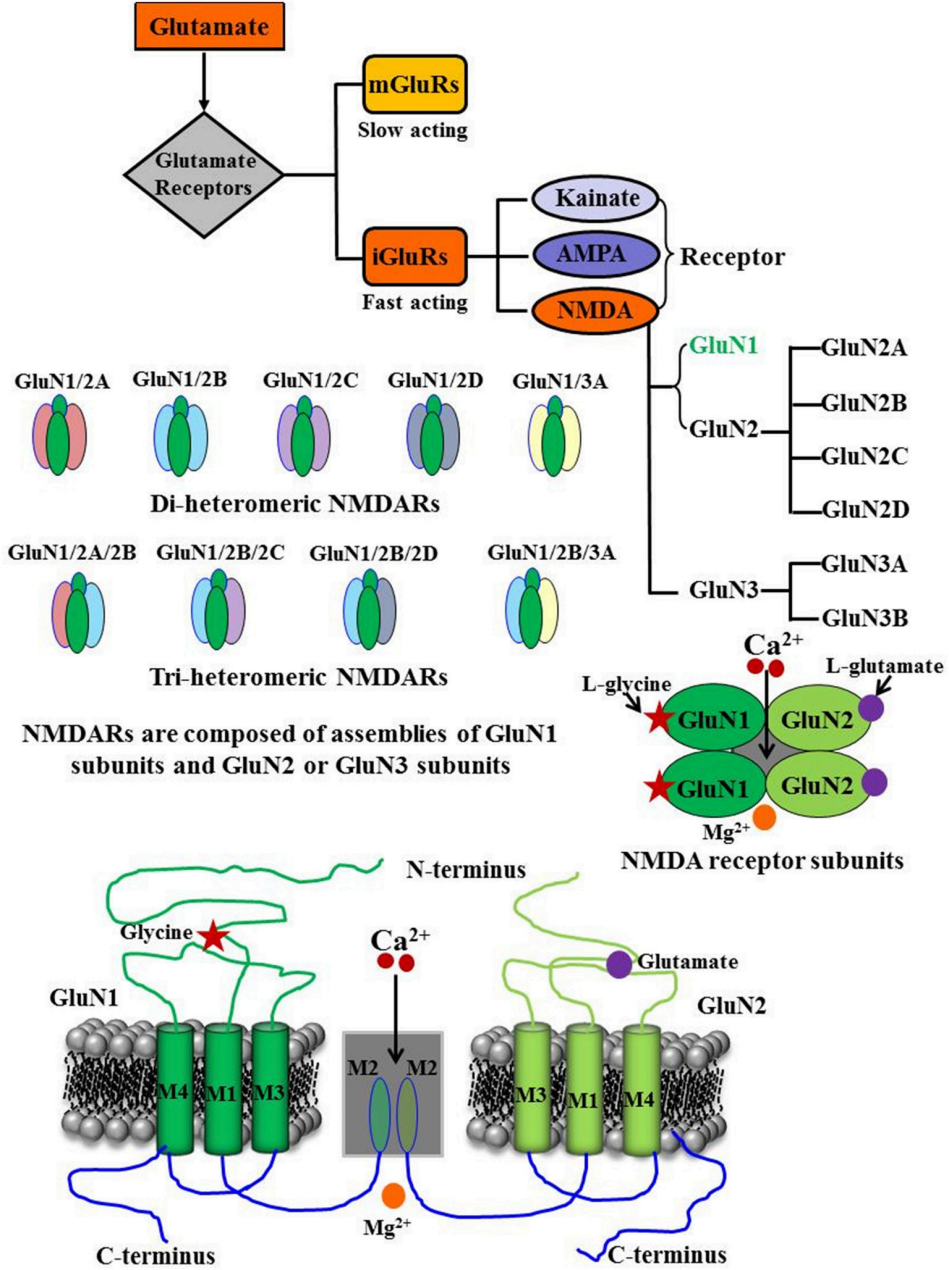
**Schematic model of the NMDA receptor and its subunit configuration**. NMDA receptors represent one of the inotropic glutamate receptors, which are composed of assemblies of GluN1 subunits and GluN2 and or GluN3 subunits. Functional NMDA receptors are composed of two GluN1 subunits and two GluN2A-D subunits; GluN3 subunits (GluN3A and GluN3B), without involving other GluN2 subunits, can assemble with GluN1 subunits to form active receptor. GluN1 subunits carry the co-agonist glycine binding site while glutamate binds to GluN2 subunit. Mg^2+^ blocks the Ca^2+^ permeable pore. All of these subunits of NMDA receptor share a common membrane topology, a large extracellular amino-terminal domain, three transmembrane segments (M1, M3, and M4), a re-entrant pore loop (M2), and an intracellular cytoplasmic C terminal domain.

N-methyl-D-aspartate (NMDA) receptors represent one of the ligand-gated non-selective ionotropic glutamate receptors (iGluRs), which are present in high density within the hippocampus and the cerebral cortex and play pivotal physiological and pathophysiological roles in the central nervous system (Cotman and Monaghan, 1989; Cotman et al., 1989). NMDA receptors along with other iGluRs, such as α-amino-3-hydroxy-5-methylisoxazole-4-isoxazopropionic acid (AMPA) and Kainate, are critical for the rapid regulation of synaptic plasticity including long-term potentiation and long-term depression, which are important cellular correlates for learning and memory function (Morris et al., 1986; Collingridge, 1987; Mondadori et al., 1989; Morris, 1989; Mondadori and Weiskrantz, 1993; Lisman et al., 1998; Martin et al., 2000). Recently, the International Union of Pharmacology Committee on Receptor Nomenclature and Drug Classification has adopted and published new guidelines to standardize the nomenclature and classification of NMDA receptor subunits (Collingridge et al., 2009). We will use this recent nomenclature to refer to various NMDA receptor subunits. These receptors are hetero-tetrameric protein complexes composed of two classes of related subunits from seven homologous genes, GluN1, GluN2A-GluN2D, and GluN3A-GluN3B (Moriyoshi et al., 1991; Kutsuwada et al., 1992; Meguro et al., 1992; Monyer et al., 1992; Laube et al., 1998; Dingledine et al., 1999; Cull-Candy et al., 2001; Figure 1).

The majority of NMDA receptors are assemblies of two GluN1 subunits, the ubiquitously expressed and obligatory subunit, and two GluN2A-D subunits, a modulatory subunit. In addition, GluN3 subunits (GluN3A and GluN3B), without involving GluN2 subunits, can assemble with GluN1 subunits to form functional receptors (Sucher et al., 1995; Laube et al., 1998; Al-Hallaq et al., 2002; Schüler et al., 2008; Low and Wee, 2010). All NMDA receptor subunits share a common membrane topology: a large extracellular amino-terminal domain, three transmembrane segments (M1, M3, and M4), a re-entrant pore loop (M2), and an intracellular cytoplasmic C terminal domain. The re-entrant M2 loop is part of the channel pore, which mediates the magnesium blockade and determines calcium permeability of the channel (Figure 1; Hollmann and Heinemann, 1994; Dingledine et al., 1999; Madden, 2002). For more details about the structure of NMDA receptor subunits, readers are requested to consult excellent review articles published recently (Magnusson et al., 2010; Traynelis et al., 2010; Magnusson, 2012; Monaghan et al., 2012; Flores-Soto et al., 2013; Sanz-Clemente et al., 2013; Wyllie et al., 2013; Shipton and Paulsen, 2014; Burnashev and Szepetowski, 2015; Glasgow et al., 2015; Zhu and Paoletti, 2015).

The activation of NMDA receptor requires binding of a ligand (glutamate) to the GluN2 subunits, membrane depolarization to remove the Mg^2+^ block of the channel, and binding of an essential co-agonist, glycine to the GluN1 subunits. For maximal activation of the NMDA receptor, binding of both glutamate and glycine are thought to be required. Results have demonstrated that D-serine might represent another physiological co-agonist of the NMDA receptor as it can bind at the glycine-binding site (Hood et al., 1989; Priestley et al., 1995; Mothet et al., 2000; Panatier et al., 2006; Labrie and Roder, 2010). NMDA receptors have slow gating kinetics (Lester and Jahr, 1990). The GluN2A-containing NMDA receptors have higher open channel probability and faster deactivation rate than GluN2B-containing receptors (Vicini et al., 1998; Chen et al., 1999; Erreger et al., 2005; Erreger and Traynelis, 2005; Gray et al., 2011). Since NMDA receptor is a non-selective cation channel, its activation and opening leads to simultaneous influx of Na^+^ and Ca^2+^ ions and efflux of K^+^ ions (Dingledine et al., 1999; Chen et al., 2005). However, between the two predominant ionotropic glutamate receptors subtypes, AMPA and NMDA, the NMDA receptors are the most permeable to Ca^2+^ ions, and the influx of Ca^2+^ ions contributes to the numerous physiological and pathological actions of the NMDA receptor (Garaschuk et al., 1996).

## NMDA receptors and senescence

**Figure 2:**
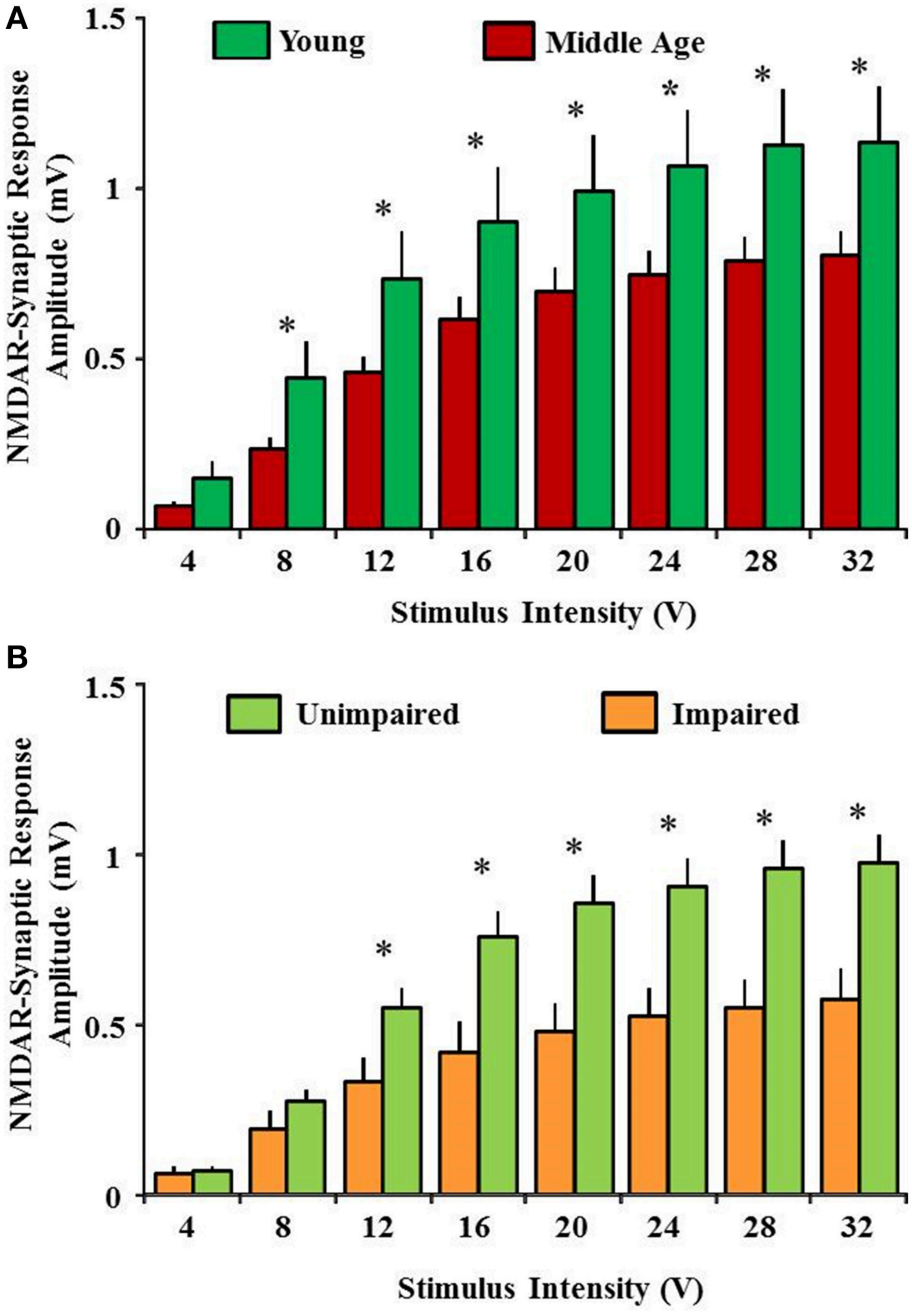
**Reduced NMDA receptor-mediated synaptic response with advanced age is associated with impaired cognitive function. (A)** Input-output curves for the NMDA receptor-mediated synaptic potentials recorded from CA3-CA1 hippocampal synapse in slices obtained from young (green) and middle age (red) animals. An age-related decrease in the synaptic response was observed. **(B)** A significant decrease in NMDA receptor-mediated synaptic response was observed in impaired animals (yellow) when compared with unimpaired (lime green). Cognitive performance was assessed by using Morris swim task. The NMDA receptor-mediated component of synaptic transmission (NMDAR-fEPSP) was obtained by incubating slices in artificial cerebrospinal fluid containing low Mg^2+^ (0.5 mM), 6,7-dinitroquinoxaline-2,3-dione (30 μM), and picrotoxin (10 μM). Input-output curves for the NMDAR-fEPSP amplitude (mV) were constructed for increasing stimulation intensities. ^*^ indicates significant difference.

NMDA receptors containing GluN2A and GluN2B subunits are highly expressed in the hippocampus and cerebral cortex (Watanabe et al., 1993a,b; Laurie and Seeburg, 1994; Monyer et al., 1994; Laurie et al., 1997; Magnusson, 2000; Magnusson et al., 2007). There is differential spatiotemporal expression and distribution of the various NMDA receptor subunits within the brain suggesting the presence of multiple NMDA receptor populations. The GluN2B subunit is highly expressed throughout the brain during early stages of development and declines at the onset of sexual maturity; GluN2A subunit-containing NMDA receptors increase across the same life span (Laurie and Seeburg, 1994; Monyer et al., 1994; Laurie et al., 1997; Law et al., 2003a,b; Liu et al., 2004). Currently, it is not well known how the multiple subunits of NMDA receptors change with advancing age and how this change may influence the cognitive function. However, evidence is mounting to indicate that advanced age is associated with a decline in NMDA receptor function and subunit expression within brain regions involved in higher brain function including synaptic plasticity, learning and memory (Gonzales et al., 1991; Pittaluga et al., 1993; Barnes et al., 1997; Magnusson, 1998a; Eckles-Smith et al., 2000; Gore et al., 2002; Liu et al., 2008a; Zhao et al., 2009). Possibly the strongest evidence for impairment in NMDA receptor function comes from physiological studies indicate the NMDA receptor mediated excitatory post-synaptic potentials in the Schaeffer collateral pathway of the hippocampus are reduced by approximately 50–60% in aged animals (Figure 2; Barnes et al., 1997; Eckles-Smith et al., 2000; Billard and Rouaud, 2007; Bodhinathan et al., 2010a; Brim et al., 2013; Kumar and Foster, 2013; Lee et al., 2014). Most recently, a robust decrease in NMDA receptor mediated synaptic function was also reported in the medial prefrontal cortex of middle aged rats (Guidi et al., 2015a). However, age-related changes in the amplitude of NMDA-evoked responses were not observed in dissociated cortical neurons suggesting the possibility of regional specificity in the loss of NMDA receptor function over the life span (Kuehl-Kovarik et al., 2003).

A decrease in the level of NMDA receptor protein expression in the hippocampus during senescence has been observed (Bonhaus et al., 1990; Kito et al., 1990; Miyoshi et al., 1991; Tamaru et al., 1991; Wenk et al., 1991; Magnusson, 1995; Magnusson et al., 2006; Billard and Rouaud, 2007; Das and Magnusson, 2008; Liu et al., 2008a; Zhao et al., 2009); further, the decrease has primarily been localized to region CA1 of the hippocampus (Magnusson and Cotman, 1993; Gazzaley et al., 1996; Magnusson, 1998a; Wenk and Barnes, 2000). These studies report reduced binding of [^3^H] glutamate (agonist site), [^3^H] glycine (GluN1 site), [^3^H] CPP (a competitive antagonist to the L-glutamate binding site), and [^3^H] MK-801 (an open channel blocker) in the hippocampus and cerebral cortex of aged rats. However, others have reported no age-related change in antagonist binding (Kito et al., 1990; Miyoshi et al., 1991; Araki et al., 1997; Shimada et al., 1997) or an increased MK-801 binding in animals with learning and retention deficits (Ingram et al., 1992; Topic et al., 2007). It is interesting to note that MK-801 binds to the hydrophobic channel domain of NMDA receptor, exclusively labeling open channels. Thus, an apparent increase in NMDA receptor channel open time may act as a compensatory mechanism for the decrease in receptor number (Serra et al., 1994; Kumar et al., 2009). However, the majority of reports, including our recent findings, indicate that the net function of the NMDA receptors decreases at CA3-CA1 hippocampal synaptic contacts during senescence (Bodhinathan et al., 2010a; Brim et al., 2013; Kumar and Foster, 2013; Lee et al., 2014). Most recently, we demonstrated a similar decline in NMDA receptor-mediated synaptic response in the prefrontal cortex of advanced age animals (Guidi et al., 2015a).

## Mechanisms for impaired NMDA receptor function during aging

### Subunit expression and composition

Alteration in expression of specific NMDA receptor subunits might be a potential mechanism for the observed decrease in the NMDA receptor function (Magnusson, 2000). Developmentally, the expression of GluN1, GluN2B, and GluN3A decreases with age compared to adulthood, while an increase in the expression of GluN2A and GluN3B is reported during development (Low and Wee, 2010). A decrease in the expression of GluN1 protein (Eckles-Smith et al., 2000; Mesches et al., 2004; Liu et al., 2008b) and GluN1 mRNA (Adams et al., 2001) levels in the aged hippocampus has been reported. The amount of C2 splice variants of GluN1 decline in the hippocampus of aged rats (Clayton et al., 2002a). In contrast, other studies report no age-related decrease in GluN1 protein expression in the whole hippocampus (Sonntag et al., 2000; Zhao et al., 2009). Despite the lack of agreement concerning changes in the expression levels in the hippocampus, other brain regions exhibit a decline in GluN1 mRNA expression during aging. Indeed, senescence-related decrease in the GluN1 mRNA expression has been observed in the medial basal hypothalamus-median eminence (Gore et al., 2002), in the medial and lateral prefrontal cortices (Magnusson et al., 2005), and in the insular, orbital, and somatosensory cortices (Das and Magnusson, 2008).

Results demonstrate age-related changes in the modulatory GluN2 subunits of the NMDA receptor. A decrease in the GluN2A protein expression has been observed in the hippocampus (Sonntag et al., 2000; Liu et al., 2008a), which is not observed in the frontal cortex (Sonntag et al., 2000). Furthermore, GluN2A mRNA expression was reported to decline in the ventral hippocampus (Adams et al., 2001). In contrast, other studies report no significant change in the GluN2A protein expression levels in the hippocampus and cortex (Sonntag et al., 2000; Martínez Villayandre et al., 2004). GluN2B subunit of the NMDA receptor is most affected by the aging process (Magnusson, 2000; Ontl et al., 2004; Magnusson et al., 2007; Zhao et al., 2009); the expression of GluN2B protein (Clayton and Browning, 2001; Mesches et al., 2004; Zhao et al., 2009) and GluN2B mRNA (Adams et al., 2001; Clayton and Browning, 2001; Magnusson, 2001) decline in the hippocampus with advanced age. This effect may be region specific since a decline in GluN2B protein is not observed in the frontal cortex (Sonntag et al., 2000). In contrast, GluN2B mRNA decreases in the frontal cortices of aging macaque monkeys, but not in the hippocampus (Bai et al., 2004). Age-associated changes in the expression of GluN2C and GluN2D of the NMDA receptor subunit are not observed in the mouse hippocampus or cortex (Magnusson, 2012), and there are few studies performed to detect the influence of aging on expression of GluN3A and GluN3B subunits of the NMDA receptor.

From a physiological point of view, the alterations in the expression of specific GluN2 subunits could have dramatic influences on NMDA receptor function through the regulation of mean channel open time and conductance of the NMDA receptors. Studies on recombinant NMDA receptor expressed in *Xenopus* oocytes demonstrate that NMDA receptors containing the GluN2A subunit (GluN2A-NMDA receptors) have faster deactivation kinetics and higher open probability relative to GluN2B containing NMDA receptors (GluN2B-NMDA receptors) (Vicini et al., 1998; Cull-Candy et al., 2001; Erreger et al., 2005; Gray et al., 2011), such that smaller ion flux is observed for the GluN2A-NMDA receptors, relative to the GluN2B-NMDA receptors. Thus, a shift in the level of GluN2 subunit expression could modulate the time course and magnitude of the Ca^2+^ signal leading to reduced Ca^2+^ influx associated with the loss of GluN2B subunits. A shift in GluN2A and GluN2B expression is thought to contribute to developmental changes in cognition and synaptic function (Dumas, 2005). Overview of literature suggests that an adequate balance between GluN2A and GluN2B might be a key mechanism for the optimal functioning of the NMDA receptor.

### Location

Alternatively, alterations in the NMDA receptor localization, through the insertion of receptors into the membrane or recruitment of extra-synaptic receptors into the synapse, may influence NMDA receptor function with advanced aging. Results suggested that GluN2B containing receptors may be more prevalent at extra-synaptic sites (Massey et al., 2004), which could temporarily house the NMDA receptors before being internalized into the cytoplasm (Blanpied et al., 2002; Lau and Zukin, 2007). In the frontal cortex, the expression of the GluN2B subunit is reduced in the synaptic membrane fraction, but not in the whole homogenate of brain tissue from senescent mice suggesting that GluN2B containing receptor sequestration at the extra-synaptic sites may be the mechanism by which the GluN2B levels decline during aging (Zhao et al., 2009). Finally, results indicate that extra-synaptic NMDA receptors couple to different signaling cascades, initiate mechanisms that oppose synaptic potentiation, by shutting off the activity of cAMP response element binding protein and decreasing expression of brain-derived neurotropic factor (Hardingham et al., 2002; Vanhoutte and Bading, 2003). However, it remains to be determined whether alteration in NMDA receptor location, specifically extra-synaptic localization, is a contributing mechanism to NMDA receptor hypofunction during senescence.

### Translational modifications

An additional probable candidate mechanism for regulating NMDA receptor function during aging is post-translational modification of the receptor. In particular, the function of the NMDA receptor is influenced by its phosphorylation state. Activation of the tyrosine kinase (Wang et al., 1994; Heidinger et al., 2002), protein kinase C (Ben-Ari et al., 1992; Chen and Huang, 1992) and protein kinase A (Raman et al., 1996) increases NMDA receptor mediated currents. In contrast, protein phosphatases, including calcineurin and protein phosphatase 1, decrease NMDA receptor currents (Lieberman and Mody, 1994; Wang et al., 1994; Raman et al., 1996). Phosphorylation state of GluN1, GluN2A, or GluN2B subunits can rapidly regulate surface expression and localization of the NMDA receptors (Gardoni et al., 2001; Chung et al., 2004; Hallett et al., 2006; Lin et al., 2006). For example, phosphorylation of serine residues within the alternatively spliced cassettes of the C-terminal tail of GluN1 promotes receptor trafficking from the endoplasmic reticulum and insertion into the post-synaptic membrane (Scott et al., 2001; Carroll and Zukin, 2002). Finally, increased phosphatase activity has been linked to the internalization of NMDA receptors (Snyder et al., 2005). Thus, the kinases and phosphatases act like molecular switches that increase or decrease NMDA receptor function, respectively. Interestingly, aging is associated with a shift in the balance of kinase/phosphatase activity, favoring an increase in the phosphatase activity (Norris et al., 1998; Foster et al., 2001; Foster, 2007). Thus, age-associated alterations in the phosphorylation state of the NMDA receptor might contribute to the decrease in the NMDA receptor function during aging (Coultrap et al., 2008). Future research is desired to delineate the direct interaction between the age-induced altered kinase/phosphatase activity and NMDA receptor function.

### Oxidative stress

**Figure 3:**
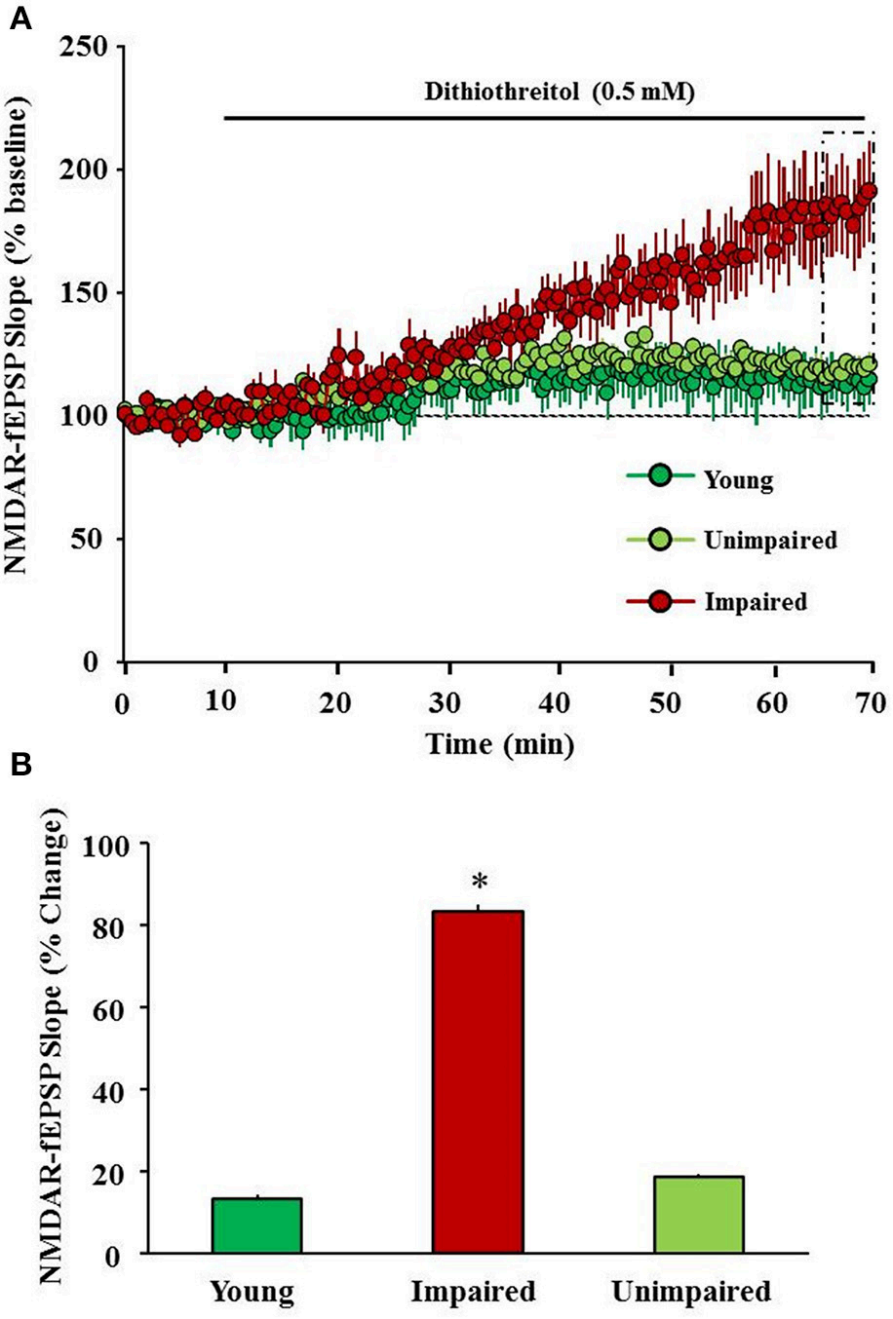
**Redox environment contributes to a decline in NMDA receptor function associated with cognitive aging. (A)** Time course of changes in the slope of NMDAR-fEPSP obtained from hippocampal slices 10 min before and 60 min after bath application of a reducing agent, dithiothreitol (DTT, 0.5 mM, solid line) for young (green circles), unimpaired (lime green circles), and impaired (red circles) animals. **(B)** Bar diagram demonstrating percent change in NMDAR-mediated synaptic response following DTT application (5 min of last 60 min) in slices obtained from young (green), unimpaired (lime green), and impaired (red) animals. DTT induced an increase in NMDAR-mediated synaptic response was significantly higher in slices obtained from impaired animals compared to young and unimpaired animals. ^*^ indicates significant difference.

Age-associated augmented oxidative stress might influence the subunit composition, expression, trafficking, and NMDA receptor function. Oxidation and reduction of sulfhydryl moieties alter NMDA receptor function. Three pairs of cysteine residues located within the N-terminal regulatory domain of the receptor (two pairs reside in GluN1 and one pair resides in GluN2A subunit) are involved in oxidation-reduction (redox) modulation of NMDA receptor (Choi and Lipton, 2000; Choi et al., 2001; Lipton et al., 2002). Previous research demonstrates that oxidizing agents such as 5,5′-dithiobis-(2-nitrobenzoic acid) (Aizenman et al., 1989), hydroxyl radicals generated by xanthine/xanthine oxidase (Aizenman, 1995) and oxidized glutathione (Sucher and Lipton, 1991) decrease NMDA receptor function in the neuronal cell cultures. The decrease in NMDA receptor function under oxidizing conditions is thought to result from the formation of disulfide bonds on the sulfhydryl group-containing amino acid residues in the NMDA receptor (Aizenman et al., 1990; Sullivan et al., 1994; Choi et al., 2001). The aging brain is associated with an increase in oxidative stress and/or a decrease in redox buffering capacity (Foster, 2006; Poon et al., 2006; Parihar et al., 2008), conditions that promote a decrease in NMDA receptor function. Results demonstrate that an age-related decrease in NMDA receptor function is related to oxidative stress and a post-synaptic shift in the intracellular redox environment (Bodhinathan et al., 2010a,b; Robillard et al., 2011; Haxaire et al., 2012; Lee et al., 2014). Our recent results provide evidence for a link between the redox-mediated decline in NMDA receptor function and the emergence of an age-related cognitive phenotype with impairment in the rapid acquisition and retention of novel spatial information (Figure 3; Kumar and Foster, 2013; Lee et al., 2014).

### Microglia interaction

Local supporting cells, astrocytes and microglia, can regulate NMDA receptor function in neurons and may provide additional possible mechanisms for the age-related changes to NMDA receptor function. Astrocytes are a major source of D-serine, an endogenous co-agonist for the NMDA receptor that binds to the glycine binding site (Schell et al., 1995). An age-related loss of D-serine is observed in the hippocampus and cortex of rats (Williams et al., 2006). Microglia contributes to the brain's immune system and activated microglia can release D-serine (Wu and Barger, 2004; Wu et al., 2004). In accordance with this idea, recent reports suggest that microglia can potentiate the NMDA receptor-mediated synaptic responses in cortical neurons (Moriguchi et al., 2003; Hayashi et al., 2006). Markers of neuroinflammation increase with advancing age and in certain neurodegenerative disorders. Finally, there is evidence for a feedback reduction in NMDA receptors due to excess synaptic glutamate activity during microglial activation (Rosi et al., 2004, 2006).

In light of the interaction of NMDA receptors and microglia, it is imperative to consider the probability that the reduction in NMDA receptor function might represent a compensatory neuroprotective mechanism associated with inappropriate receptor activity or increased Ca^2+^ due to other mechanisms. Thus, impaired NMDA receptor-dependent synaptic plasticity and memory decline may be epiphenomena due to processes for cell preservation (Foster, 1999). Indeed, over expression of GluN2B subunits improves synaptic plasticity and memory in aged mice (Cao et al., 2007; Brim et al., 2013) indicating that increased NMDA receptor function can rescue physiological symptoms of cognitive aging. However, cognition and synaptic plasticity are also improved by treating with the low-affinity voltage-dependent NMDA receptor channel blocker, memantine (Barnes et al., 1996; Pietá Dias et al., 2007), possibly by reducing inappropriate NMDA receptor activity (Rosi et al., 2006; Matute, 2007; Chang and Gold, 2008). Memantine, a non-competitive moderate and partial NMDA receptor channel blocker, can attenuate the over activation of the NMDA receptor by preventing influx of excessive Ca^2+^ without influencing physiological activity of NMDA receptor (Scarpini et al., 2003; Gardoni and Di Luca, 2006; Pallàs and Camins, 2006). In clinical studies, memantine has been demonstrated to have beneficial effects for patients with moderate to severe Alzheimer's disease, while has no effect in mild AD patients (Reisberg et al., 2003). Initial studies described positive effects of memnatine on cognition in demented patients (Ditzler, 1991), neuronal plasticity and learning in senescent animals (Barnes et al., 1996), water maze performance in adult male rats (Zoladz et al., 2006), and spatial learning in transgenic mice (Minkeviciene et al., 2004). However, results for the efficacy of memantine's beneficial influence on cognition are controversial. Memantine reduces agitation and delusions in severe AD cases and yet has little or no cognitive benefit for mild AD, including impaired executive function (Reisberg et al., 2003; Parsons et al., 2007; Schneider et al., 2011; Huang et al., 2012). Furthermore, a recent study demonstrated that memantine fail to improve significant agitation in people with moderate to severe AD (Fox et al., 2012). Future studies are required to clearly examine the efficacy of memantine for cognitive benefits and other neuropsychiatric symptoms.

## NMDA receptor hypofunction and its influence on cognitive performance

NMDA receptors play an important role in learning and memory, and are critical for spatial memory (Morris et al., 1986; Collingridge, 1987; Mondadori et al., 1989; Morris, 1989; Mondadori and Weiskrantz, 1993; Lisman et al., 1998; Martin et al., 2000; Foster, 2012; Guidi et al., 2015b); NMDA receptor-mediated synaptic plasticity and its hypofunction is thought to play a vital role in age-associated cognitive impairments (Foster and Norris, 1997; Foster, 2012; Kumar and Foster, 2013). Advanced age is associated with a decrease in subunit expression and a decline in NMDA receptor function. Mounting evidence suggests that an age-associated deficiency in NMDA receptor contributes to impairment in spatial learning and memory (Davis et al., 1993; Magnusson, 1998b, 2001; Clayton et al., 2002b; Magnusson et al., 2007; Das and Magnusson, 2008, 2011; Zhao et al., 2009; Das et al., 2012; Foster, 2012; Zamzow et al., 2013). The age-associated decrease in the GluN2B subunit of the NMDA receptor contributes to some forms of memory decline during senescence (Zamzow et al., 2013). Prior work regarding the NMDA receptor and its functional state during aging has been largely focused on the hippocampus (Barnes et al., 1997; Magnusson, 1998a; Eckles-Smith et al., 2000; Zhao et al., 2009; Kumar and Foster, 2013). Our results also provide evidence by showing blunted NMDA receptor mediated synaptic potentials in memory impaired animals when compared with unimpaired animals (Figure 2B; Kumar and Foster, 2013). These results demonstrate that NMDA receptor-mediated synaptic responses at CA3-CA1 hippocampal synapses are significantly decreased with advanced age. Examination of synaptic transmission, according to behavioral classification, revealed that animals classified as impaired exhibited a decrease in the NMDA receptor component of the synaptic response relative to young and unimpaired animals. Recent results successfully demonstrate that viral vector mediated upregulation of the GluN2B subunit of NMDA receptor in aged animals enhances hippocampal-dependent memory and synaptic transmission (Brim et al., 2013). Furthermore, we provide evidence for a link between the redox-mediated decline in NMDA receptor function and the emergence of age-related memory impairment (Kumar and Foster, 2013). These results demonstrate that bath application of the reducing agent dithiothreitol enhanced the NMDA receptor component of the synaptic response to a greater extent in impaired animals relative to unimpaired and young rats (Figure 3).

In addition to the hippocampus, there are studies that indicate an age-associated decrease in NMDA receptor-mediated synaptic plasticity in other brain regions that might contribute to impairments in cognitive function (Bourne and Harris, 2007; Morrison and Baxter, 2012). Our most recent results demonstrate a robust decrease in NMDA receptor mediated synaptic responses in the medial prefrontal cortex, and the decrease in NMDA receptor function was due in part to an altered redox state, which was associated with several measures of prefrontal cortex-dependent behavior (Guidi et al., 2015a). These results indicate that redox mediated changes contribute to altered NMDA receptor function and provide possible mechanisms that may underlie impairment in cognitive performance associated with advanced age. However, a clear understanding, in regards to how alterations in NMDA receptor function associated with either advanced age or with neurodegenerative diseases that contribute to cognitive impairment, is essential.

## Restoring NMDA receptor function with advanced age

One simple notion is that augmenting the NMDA receptor subunit expression and function should ameliorate cognitive function. The challenge is how to restore or prevent the diminishing NMDA receptor function over the course of a life span and in neurodegenerative diseases. Pharmacological agents such as N-acetylcysteine (NAC) and D-cycloserine have provided an avenue to enhance NMDA receptor function and reverse negative consequences associated with NMDA receptor hypofunction. NAC is a derivative of the amino acid L-cysteine with an added acetyl group attached to the nitrogen atom, which serves as a precursor for the formation of the antioxidant glutathione (γ-glutamylcysteinylglycine; GSH) in the body (Ziment, 1988). The tripeptide GSH is a potent endogenous antioxidant produced by cells and is important for the maintenance of redox potential in the brain (Reid and Jahoor, 2001; Cruz et al., 2003). The thiol (sulfhydryl) group confers the antioxidant properties. Previous research examining the ability of reducing and oxidizing (redox) agents to modulate NMDA receptor activity in cell cultures and in tissue from neonates suggests that redox state is an imperative determinant of NMDA receptor function possibly through oxidation of extracellular cysteine residues on the NMDA receptor subunits; NMDA receptors are redox sensitive protein that requires a healthy redox balance (Aizenman et al., 1989, 1990; Tang and Aizenman, 1993; Bernard et al., 1997; Choi and Lipton, 2000; Lipton et al., 2002). The redox sensitive intracellular molecules affect NMDA receptor function and the aged brain exhibits augmented oxidative damage and a decrease in redox buffering capacity (Foster, 2006; Poon et al., 2006; Parihar et al., 2008). Our published results demonstrate that age-associated enhanced oxidative stress contributes to NMDA receptor hypofunction, and the reducing agent significantly improved the NMDA receptor mediated synaptic response (Bodhinathan et al., 2010a; Kumar and Foster, 2013; Lee et al., 2014; Guidi et al., 2015a; Figure 3). NAC, being an antioxidant and anti-inflammatory agent, has the abilities to modulate NMDA receptor activity. Long-term dietary treatment with NAC is beneficial in alleviating age-associated neuronal alterations induced by an impaired antioxidant defense system (Martínez et al., 2000; Cocco et al., 2005). Results from a recent study eloquently demonstrated that long-term dietary supplementation with NAC prevented age-induced oxidative damage in the hippocampus and restored NMDA receptor-mediated long-term potentiation at CA3-CA1 hippocampal synapses. Additionally, the authors demonstrated that age-associated decrease in levels of D-serine, a NMDA receptor co-agonist that is required for receptor activation, and expression of serine racemase, an enzyme that is responsible for synthesis of D-serine from L-serine, are rescued by long-term dietary treatment with NAC (Haxaire et al., 2012). NAC, being a precursor of GSH, can protect the brain from low levels of GSH and improve cognitive performance (Fu et al., 2006; Robillard et al., 2011). Results also demonstrate that long-term dietary supplementation with NAC can restore age-induced impaired hippocampal synaptic plasticity close to that of the adult level (Robillard et al., 2011). An enhancement in NMDA receptor current has also been observed following acute application of NAC in cortical neurons (Dukoff et al., 2014). Review of the literature provides abundant evidence for beneficial influence of NAC on antioxidant balance, neurotransmission, neurogenesis, and inflammation. Adjunctive supplementations with NAC improve the symptoms of bipolar disorder, Alzheimer's disease, depression, cognitive impairment, and other psychiatric disorders (Butterfield and Pocernich, 2003; Jayalakshmi et al., 2007; Lanté et al., 2008; Choy et al., 2010; Goncalves et al., 2010; Jain et al., 2011; Otte et al., 2011; Dean et al., 2012; Dhanda et al., 2013; Rodrigues et al., 2013; Carvalho et al., 2014; Steullet et al., 2014; Thakurta et al., 2014; Soleimani Asl et al., 2015). For more details about therapeutic potential of NAC in ameliorating various pathological conditions, readers are referred to excellent review articles published previously (Kelly, 1998; Dean et al., 2012). Future studies are needed to explore the beneficial influence of NAC on NMDA receptor hypofunction and its consequence on cognitive impairments in hippocampal dependent spatial memory and prefrontal cortex-dependent executive function associated with senescence and other pathological disorders.

Another pharmacological agent, D-cycloserine ((4*R*)-4-amino-1,2-oxazolidin-3-one, DCS), a partial agonist and positive modulator of the NMDA receptor, binds at strychnine-sensitive glycine modulatory site of the receptor and enhances NMDA receptor function (Johnson and Ascher, 1987; Reynolds et al., 1987; Hood et al., 1989; Kemp and Leeson, 1993). DCS acts as an antagonist by shifting more efficient endogenous agonists like D-serine at high dose. A low dose of DCS can act as an agonist and facilitate NMDA receptor function (Horio et al., 2013). There are numerous reports suggesting the beneficial influence of DCS on cognitive performance and other neurodegenerative diseases (Thompson et al., 1992; Baxter et al., 1994; Ohno and Watanabe, 1996; Gabriele and Packard, 2007; Yaka et al., 2007; Curlik and Shors, 2011; Kuriyama et al., 2011; Feld et al., 2013; Kranjac et al., 2013; Ren et al., 2013). Peripheral injection of DCS, which crosses the blood-brain barrier, has been shown to enhance learning rate and rescue impaired memory consolidation (Thompson et al., 1992; Kranjac et al., 2013). DCS can modulate NMDA receptor-mediated neurotransmission and has the potential to restore impaired NMDA receptor function associated with aging or pathological condition. Results indicate that age-associated impaired cognition is improved by DCS treatment (Baxter et al., 1994; Aura and Riekkinen, 2000). In young animals, DCS enhanced NMDA receptor activation and facilitated hippocampal neurotransmission (Pitkänen et al., 1994; Rouaud and Billard, 2003; Donzis and Thompson, 2014). Furthermore, a senescence-induced deficit in NMDA receptor mediated neurotransmission is restored by DCS (Billard and Rouaud, 2007). A study by Kochlamazashvili and colleagues demonstrated that DCS restored learning in the fear conditioning paradigm and improved long-term NMDA-mediated synaptic transmission in aged neural cell adhesion molecule deficient animals by upregulating the GluN2A subunit of the NMDA receptor (Kochlamazashvili et al., 2012). Readers interested in details regarding to DCS influences on cognitive performance and neurotransmission over the life span and in neuropathological conditions may consult previously published review articles (Norberg et al., 2008; Huang et al., 2012; Lin et al., 2014; Hofmann et al., 2015). Future studies are needed in order to delineate the influence of DCS on specific NMDA receptor subunit configurations and its functional outcome in regards to ameliorating age-associated impaired cognition and NMDA receptor hypofunction over the life span.

D-serine is a potent endogenous ligand that binds at the glycine binding site of the NMDA receptor and acts as a neuronal signaling molecule leading to upregulation of NMDA receptors. In addition, it is released from astrocytes and is highly expressed in the brain (Schell et al., 1995; Mothet et al., 2000; De Miranda et al., 2002; Nishikawa, 2005; Fossat et al., 2012). D-serine is required for NMDA receptor activation, and may have preferential affinity/effectiveness for NMDA receptors that contain GluN2B subunits (Priestley et al., 1995). The levels of D-serine are dramatically reduced with advanced age (Junjaud et al., 2006; Mothet et al., 2006; Turpin et al., 2011). One possibility is a loss of the serine racemase enzyme, which generates D-serine from L-serine (Wolosker et al., 1999a,b). A decline in serine racemase mRNA is observed in the prefrontal cortex of aging humans (Loerch et al., 2008) and the hippocampus of aged rats (Turpin et al., 2011). The enzyme serine racemase generates D-serine from L-serine; pharmacological or viral gene delivery tools could be employed to increase endogenous levels of D-serine or serine racemase expression. Currently, there is some debate as to the source of serine racemase, either neuronal or glial (Wolosker et al., 1999a; Wolosker, 2011; Wolosker and Mori, 2012); however, recent results provide convincing evidence that serine racemase is primarily expressed by neurons (Yoshikawa et al., 2007; Miya et al., 2008; Rosenberg et al., 2010; Ding et al., 2011). There are reports that oral treatment or intracerebroventricular infusion of DCS induced a marked increase in extracellular D-serine levels within the hippocampus of *SRR*-knockout mice (Horio et al., 2013). Future studies to upregulate the expression of serine racemase, in order to enhance the endogenous level of D-serine, could provide another avenue to restore impaired NMDA receptor function during aging and under pathological conditions.

Recent studies, employing genetic tools, provide another alternative to enhance NMDA receptor function. A great advantage of this technology is that we can study the effect of single genes, or gene combinations by injecting mixtures of viral vectors to upregulate expression of target genes. Dose response studies, by injecting dilutions of viral vector, to analyze the effects of different levels of expression/transduction of the GluN1/GluN2A/B subunits, can also be performed along with functional significance of enhanced expression of the receptor. Viral vectors were originally developed as an alternative to transfection of genetic material (DNA) into cells. Now the viral vector-mediated gene delivery technique is more commonly used to upregulate or downregulate a target gene into living cells. This tool provides an excellent opportunity to overexpress various subunits of the NMDA receptor and measure its functional consequence on behavioral performance. Our results clearly illustrate rescuing age-associated impaired hippocampal-dependent spatial memory by enhanced expression of the GluN2B subunit of the NMDA receptor (Brim et al., 2013). More research in this direction will be essential to explore the impact of augmenting expression of various NMDA receptor subunits alone or in combinations on NMDA receptor function including synaptic transmission and cognitive performance. In addition to analyzing beneficial influence of augmented NMDA receptor subunit expression/function, future research should also investigate possible side effects and other stipulations associated with enhanced function of NMDA receptor.

## Conclusion and outlook

Now there is mounting evidence, which suggest that the subunit expression, configuration, and function of the NMDA receptor are altered with advancing age. Vista of factors including subunit expression, topography, oxidative stress, interaction with scaffolding proteins and glial cells, receptor trafficking, and translation, tempered by aging might be contributing to NMDA receptor hypofunction. Due to the critical importance of NMDA receptors in synaptic transmission and memory, a selective upregulation of NMDA receptor subunits in neurons may provide an avenue for treating age-associated cognitive deficits. Clearly, future research will need to delineate the contributions of several mechanisms in optimizing specific subunit contribution and influence of upregulation in mediating learning and memory function. Thus, it will be imperative for future research to determine whether enhancing or inhibiting NMDA receptor function by upregulating or downregulating different subunits expression configurations will be beneficial in preserving cognitive domain during normal senescence.

### Conflict of interest statement

The author declares that the research was conducted in the absence of any commercial or financial relationships that could be construed as a potential conflict of interest.
